# How do novice and improver walkers move in their home environments? An open-sourced infant’s gait video analysis

**DOI:** 10.1371/journal.pone.0218665

**Published:** 2019-06-20

**Authors:** Jitka Marencakova, Carina Price, Tomas Maly, Frantisek Zahalka, Christopher Nester

**Affiliations:** 1 Sport Research Centre, Faculty of Physical Education and Sport, Charles University, Prague, Czech Republic; 2 Research Centre of Health Sciences, School of Health and Society, University of Salford, Salford, United Kingdom; Universidade Federal do Rio Grande do Sul, BRAZIL

## Abstract

**Objective:**

Natural independent walking mostly occurs during infant´s everyday explorations of their home environment. Gait characteristics of infant walkers at different developmental stages exist in literature, however, data has been only collected in laboratory environments, which may reduce gait variability, therefore mask differences between developmental stages of natural gait. The aim of the study was to provide the first data set of temporal and functional gait characteristics of novice and improver infant walkers in familiar environment conditions in their home. We hypothesised that familiar environment conditions may effectively demonstrate natural gait characteristics and real differences in gait variables differing between 2 groups of developing infant walkers.

**Methods:**

In a cross-sectional design; we used open-source videos of infants in their home environments: twenty videos of 10 novice (5 girls, 5 boys, 7–12 months) and 10 improver (4 girls, 6 boys, 8–13 months) walkers were chosen from an open-source website. 2-D video gait analysis was undertaken for these parameters: falls frequency, frequency of stops, gait cadence, and time of stance phase, swing phase, and double support. Between groups comparison for novice versus improver was investigated by Mann-Whitney U tests (*p* ≤ 0.05) with determination of effect size of Pearson *r* correlation.

**Results:**

Statistically significant differences between groups with large effect sizes were found for these parameters: falls frequency (*p* = 0.01, *r* = 0.56); cadence (*p* = 0.01, *r* = 0.57); stance phase duration of right leg (*p* < 0.01, *r* = 0.63); stance phase duration of left leg (*p* = 0.01, *r* = 0.56); and double support phase duration (*p* < 0.01, *r* = 0.69). Novices scored higher in comparison with improver walkers in all the parameters except cadence.

**Conclusions:**

This study presents the first data set of functional and temporal gait parameters of novice and improver infant walkers in their home environments. As an addition to recent research, novice infants walk with lower cadence and higher falls frequency, stance phase time and double support in their familiar environments. With increasing experiences, infant´s cadence increases while the other parameters decrease.

## Introduction

Ontogenesis of walking starts very early in human development, even intra-uterine at the nervous system level [1]. Just after birth, first pre-mature infant stepping can be evoked when a baby is held in an erect position with their feet touching the ground. As the maturation process continues, the phase of verticalization to upright posture in gravity and bipedal supported walking follow with gradual development into independent walking [2,3].There are two phases of independent walking maturation identified in literature [4–7]. The first phase, lasting from 3 to 6 months after the onset of independent walking, is devoted to the learning of gait postural requirements in gravity [7]. The second phase, lasting several years, involves fine tuning of gait [4–6].

Characteristics of the early stage of walking show a large inter- and intra-individual variability and variable walking maturation strategies: for example twister, faller, stepper, pendulum [8]. Despite this variation, there are some typical patterns within this early stage of walking reported in literature; Hallemans et al. describe that foot contact when walking in gait laboratory in novice walker is undertaken in three different patterns: (i) heel strike, (ii) flat-foot contact, and (iii) toe walking until 22 weeks of walking experience [9,10]. Furthermore, novice walkers specifically walk with wider base of support [5] and higher lift of leg and foot during swing phase [11] in gait laboratory protocols. Some authors added that the classic pendulum mechanism is not yet implemented in novice walking infants [5], developing only after a few months of walking experience and is evident in more improved walking infants [8]. During this early stage the upper body and trunk is unstable [6] and the upper arms are typically carried up in a high guard position above their centre of mass. This allows early walkers to use these segments to counter the over- or under-rotation of the trunk and head as the body passes through space [1]. The position of the arms drops as walking experience develops [12]. Early walking infants are more uncertain with an obvious higher frequency of falls compare to more mature independent walkers [13], however the prevalence of falls in more familiar environments in novice or improver infants is not documented. We may anticipate novice walker infants in particular to be less functionally competent at walking in less familiar environments.

Natural walking of infants mostly occurs during their everyday explorations of environment and interactions with their parents and caregivers [13]. Whilst the gait characteristics of walking infants and comparisons of early and experienced walkers exists in literature [13–16], biomechanical data has only been collected in laboratory environments. These environments often include instrumentation frequently designed for adults and methodologies which can be prescriptive and limiting in terms of the variation in gait that might occur in less constrained settings. To obtain optimal data, researchers often have encouraged infants or have held their hand to walk through the measurement area [15], or they have excluded some part of natural sequences which they did not require for their data analysis such as hopping or running [16]. Supporting the infant to walk during laboratory data collection, imposes a direction on the infant which may not reflect their natural voluntary movements and data may not fully represent the variability of their walking pattern and therefore be of low external validity and mask differences between developmental stages. Similarly, deleting aspects of trials which include inconsistent gait patterns during progression also removes an aspect of familiar gait which may be characteristic of their current development and results in this not being described. In wider literature some approaches to record natural infant motion are evident, particularly in motor control and psychology research. Cole et al. [14] and Adolph et al. [13] use a laboratory environment designed to be familiar to infant with a large area for moving independently. Cole et al. [14] observed the bouts of steps (in terms of length and direction) in two groups: 13 and 19 months old infants. However, the data presented in these two studies still includes data in an unfamiliar and contrived environment, which is not the home environment of the infant. Other methods mentioned in literature which could be used for collecting real world data include video recording [13,17] wireless inertial sensors [8] and 3D motion capture systems [10]. Despite these systems being effective in real world conditions, all authors used them only in laboratory or clinical environments to investigate infant gait. Video recording however, offers the opportunity to record gait parameters in familiar environments and open source video has been utilised to capture real-world movements in prosthetic users [18]. Gardiner et al. [18] highlighted the advantages of obtaining less available gait data, for example from unusual amputation level amputees and/or other challenging health conditions patients outside the laboratory conditions, i.e. various ground surfaces, outside obstacles etc. Similarly, open-source videos offer the potential to collect data of infants walking in self-directed manners in their home environments, without any markers or sensors on their body, and free of experimental artefacts.

The aim of this study was to provide the first data set of temporal and functional characteristics of novice and improved infants walking in familiar environment conditions in their homes. Inspired by the study of Gardiner et al. [18] who used crowd-sourced video for analysis of amputee gait temporal characteristics, we used open source videos for our primary data. We hypothesised that data analysis of video from familiar environment conditions would be able to discriminate differences in gait variables between novice and improved walkers. We hypothesised that infant functional gait characteristics would demonstrate an improvement (such as less falls) and temporal characteristics would demonstrate increases in stability (such as decreased double support time) with experience during the gait maturation process.

## Materials and methods

### Video searching

**Fig 1:**
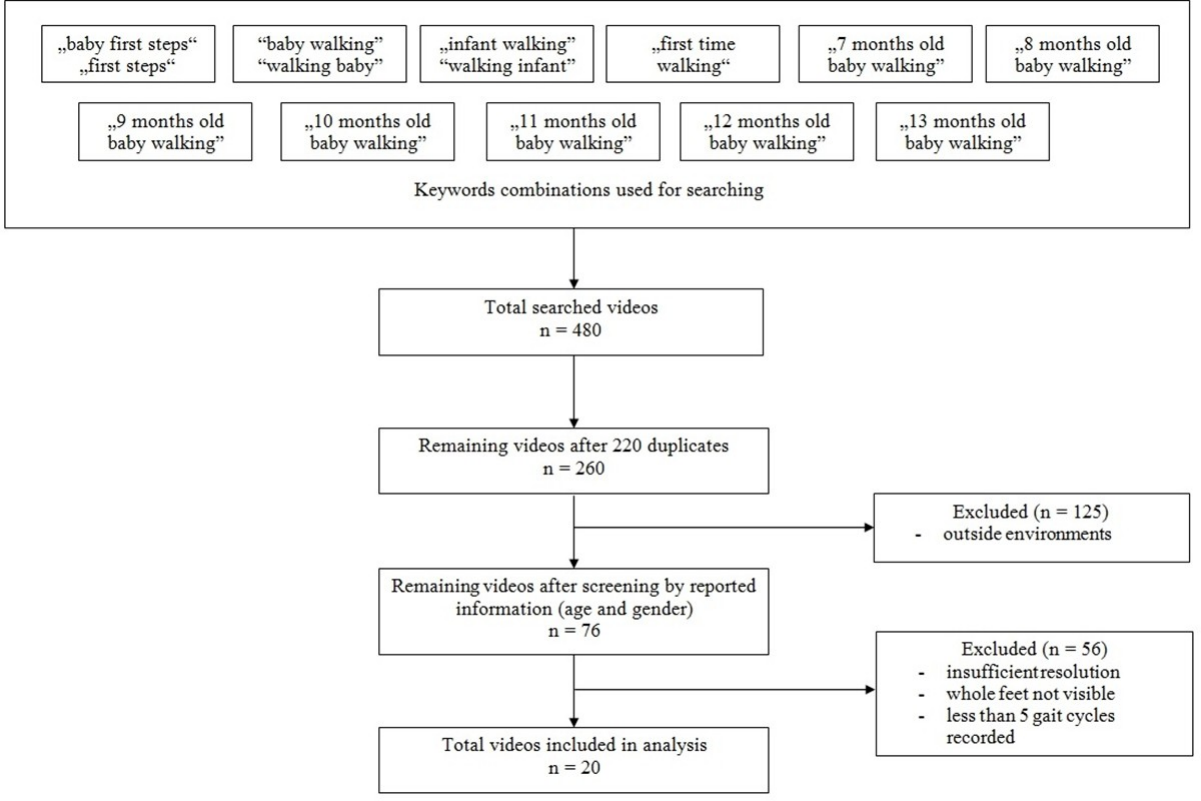
Video searching flowchart.

The open-source video sharing website YouTube (www.youtube.com) was searched for videos of walking infants in their home environments. The single researcher conducted searches of YouTube’s database between the 25^th^ of January and the 13^th^ March 2018 (Fig 1). The website was queried using the following keywords in a variety of combinations: ‘walking’, ‘baby’, ‘infant’, ‘first steps’, ‘first time’, ‘7-13months old baby walking’. A total of 480 videos were downloaded by a free video downloader (www.clipconverter.cc). Only good resolution videos that contained information about the age and gender specification of the infant, where both feet were visible to observe clear footage of independent walking on level ground and recorded a walking bout consisting of a minimum of five complete gait cycles were selected for further analysis, regardless of language or ethnicity. Videos that contained walking outside were rejected due to the potential confounding factors of wide varieties of surfaces and environments. This resulted in 20 videos being selected. All data from the YouTube´s videos were made anonymous and collected under the ‘Fair Use’ (https://www.youtube.com/yt/copyright/en-GB/fair-use.html) and ‘Exceptions to Copyright’ (https://www.gov.uk/guidance/exceptions-to-copyright) rules for non-commercial research.

### Research sample

The resulting downloaded videos were sorted by three independent investigators, blinded to video name and text relating to the participants, into 2 groups; novice and improver walker. All three investigators had at least 2 years experience in children gait analysis and all agreed unambiguously on 18 videos. The two videos where all 3 evaluators did not agree were put into the group which was selected by two of investigators. Inclusion criteria for the novice group were subjectively chosen according to existing evidence [13,19] and based on reviewer experience. Criteria included hands in high-guard position, uncertainty during walking, and a lack of stability and control. Sorting resulted in two groups of N = 10 infants; novice walkers (5 female and 5 male, 7–12 months) and improver walkers (4 female and 6 male, 8–13 months).

### Data collection

#### Gait parameters

Mean and median of selected functional and temporal gait characteristics for each infant and both groups were investigated by one single investigator: (i) functional characteristics—frequency of falls (falls per minute) and frequency of stops (stops per minute); and (ii) temporal characteristics—gait cadence (steps per minute), stance phase time (seconds) for right leg and left leg, swing phase time (seconds) for right leg and left leg, and double support phase time (seconds). Foot contact and foot off times were recorded for the both legs during whole walking sequence of each video. Foot contact was defined as a moment when the foot first visibly touched the ground. Foot off described the occasion when the foot first visibly left the ground. Stance phase time was defined as time elapsed between foot contact and the consecutive foot off the same leg. Thus, it is a summation of double support time and single leg stance time within one stride. Swing phase time was between foot off and the consecutive foot contact of the same foot and is equal to single leg stance of opposite leg. Double support time was defined as the duration elapsed during which both feet contacted the ground. A fall was defined as a sudden unintended loss of balance and collapse of the participant, down to the ground, but with evident willingness to stand up and continue to walk. A stop was defined as an intended break in walking mainly for recovery of balance or short change of the focus of infant (i.e., watching the parent, playing the carrying object, etc.). Based on our observations, the time of stop lasting less than 4 s was counted as double support phase time.

#### Video analysis

The videos were systematically analysed by one single evaluator and 2D video analysis was performed using free accessible tool Tracker 4.11.0 (www.opensourcephysics.org), which allows processing of the video footage frame by frame and show the sampling rate of the original video recording. From recorded times of foot contact and foot off, the average of stance phase and swing phase time for both legs and the double support time was calculated. The researcher recorded the age, gender, ethnicity assumption, clothing conditions, and counted number of steps, falls, and stops to calculate gait cadence and frequency of falls and stops. The original video sampling rate was recorded also, as it may influence time dependent results.

### Statistical analysis

Shapiro-Wilk test and Levene´s test were used for data investigation for normal distribution and homogeneity of variance. As the data failed the assumptions for parametric tests, non-parametric descriptive statistics (median and interquartile range IQR) were used. For inductive statistic, Mann-Whitney U test for two independent groups and comparison of mean ranks was performed due to difference in distribution of scores for both groups. Effect size of Pearson *r* correlation [20] was calculated for all variables with low effects 0.1, medium effects 0.3, and large effects 0.5 and higher [21].

All statistical tests were performed using the statistical software SPSS (IBM SPSS Statistics for Windows, Version 24.0. Armonk, NY: IBM Corp., 2016) and *p* ≤ 0.05 was set as a level of significance.

## Results

**Fig 2:**
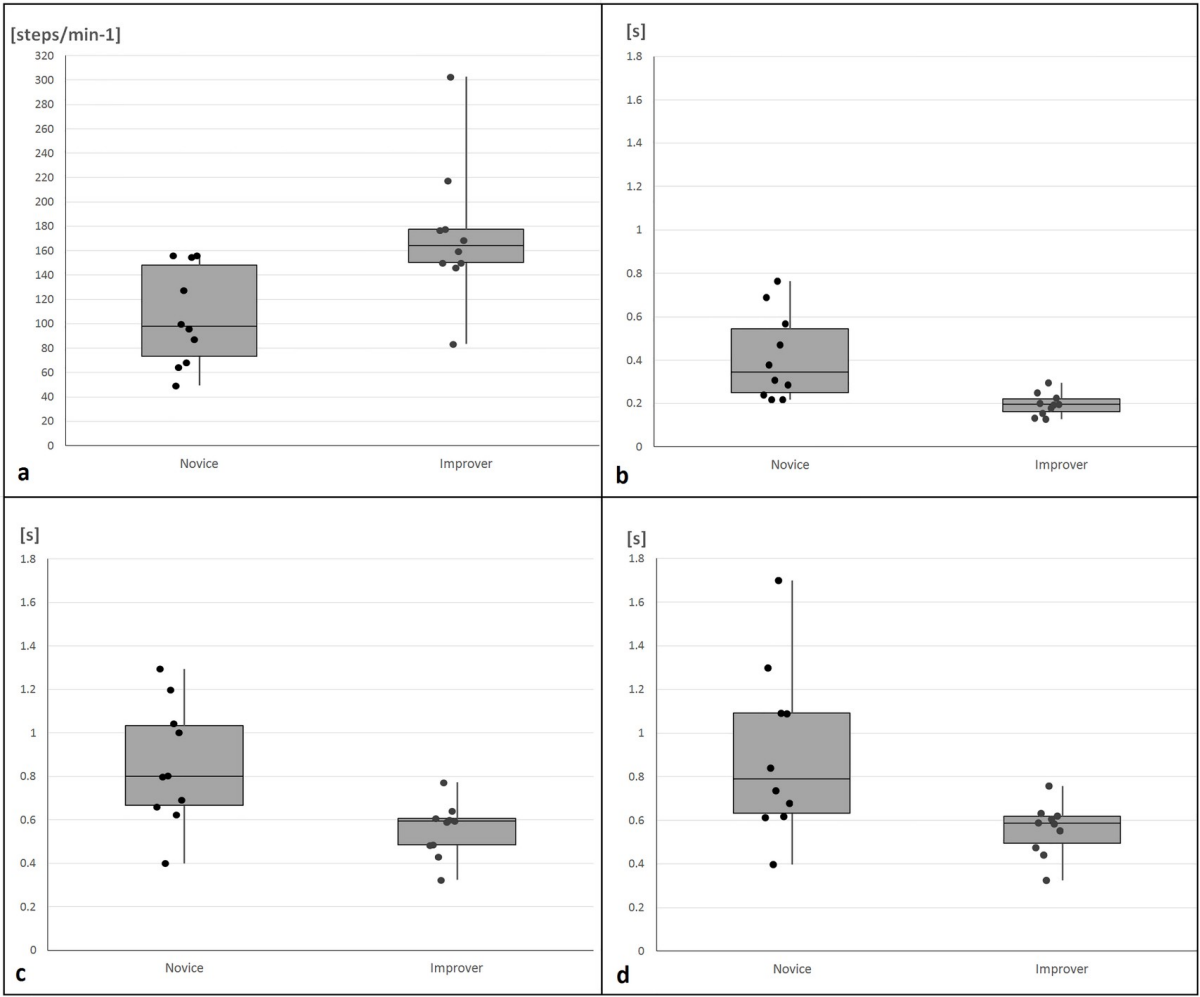
**Graphical illustrations of significantly different infant temporal gait characteristics between the novice and improver with individual distributions a) cadence b) double support time c) stance phase time right leg d) stance phase time left leg.** Where box-plots denote the median and inter-quartile ranges of the data, lines indicate the maximum and minimum data values and all data points for N = 20 infants are recorded with points.

The research sample description of novice and improver walker infants and additional external conditions are shown in Table 1. Novice and improver walker infants in home environments showed significant differences (*p* < 0.05) in functional and temporal gait characteristics. Novices scored higher for five parameters with large effect sizes: frequency of falls (FALLS: *p* = 0.01, *r* = 0.56), stance phase time for right leg (ST_R: *p* < 0.01, *r* = 0.63), left leg (ST_L: *p* = 0.01, *r* = 0.56) and double support phase time (DS: *p* < 0.01, *r* = 0.69), but lower for gait cadence (CAD: *p* = 0.01, *r* = 0.57) (Tables 2 and 3). These variables demonstrated greater inter-individual variability in novice compared to improver walkers (Fig 2).

**Table 1:** Research sample and external conditions characteristics.

Infant		Gender	Age (months)	Ethnicity	Barefoot/Shoes	Carrying object	Environment		Motivation
							Corridor/Big room/Small room	Carpet/Floor	Parent/Self-motivation
**Novice**	1	M	8	Mix	BF	No	BR	F	P
	2	F	10	W	BF	Yes	C	F	P
	3	F	7	B	BF	No	SR	F	P
	4	F	11	W	BF	No	C	F	P
	5	M	9	Mix	BF	No	C	CA	P
	6	M	8	W	S	No	SR	F	P
	7	F	9	ME	BF	No	BR	F	P
	8	F	8	W	BF	No	BR	CA	P
	9	M	7	B	BF	No	C	F	P
	10	M	12	B	S	No	C	F	P
**Improver**	1	F	12	A	BF	No	C	CA	P
	2	M	10	W	BF	Yes	BR	CA	Self
	3	F	8	W	BF	No	BR	F	Self
	4	M	11	W	S	No	BR	F	Self
	5	M	12	W	S	Yes	BR	F	P
	6	M	12	A	BF	Yes	C	F	Self
	7	F	11	W	BF	Yes	C	F	Self
	8	M	13	W	S	No	BR	CA	Self
	9	F	12	W	BF	No	BR	CA	Self
	10	M	9	B	S	No	BR	CA	Self

M, male; F, female; W, white; B, black; A, Asian; Mix, mixed.

**Table 2:** Dataset with individual infant gait data of novice N = 10 (group 1) and improver N = 10 (group 2) walkers.

Group	Sampling rates (Hz)	Duration of video sequence (s)	Number of steps	Falls frequency (per min)	Stops frequency (per min)	Cadence, CAD(steps/min)	Time of stance phase (s)			Time of swing phase (s)			Time of double support phase, DS (s)
							ST_R	ST_L	ST_Diff _ΔR-L_	SW_R	SW_L	SW_Diff _ΔR-L_	
**1**	0.033	16.133	42	0	3.719	156.202	0.660	0.614	0.045	0.135	0.181	0.046	0.238
**1**	0.033	43.486	36	4.139	1.380	49.671	1.296	1.299	0.003	0.146	0.140	0.006	0.691
**1**	0.040	16.731	18	3.586	3.586	64.551	0.798	0.842	0.044	0.104	0.086	0.018	0.379
**1**	0.067	46.113	77	10.409	1.301	100.189	0.401	0.398	0.003	0.084	0.064	0.019	0.218
**1**	0.033	9.877	21	0	0	127.569	0.805	0.737	0.068	0.210	0.191	0.020	0.307
**1**	0.033	18.400	21	0	9.783	68.478	1.199	1.700	0.501	0.171	0.128	0.043	0.764
**1**	0.034	26.862	43	0	4.467	96.046	1.043	1.093	0.050	0.168	0.151	0.017	0.473
**1**	0.033	6.200	16	9.677	0	154.839	0.624	0.619	0.004	0.183	0.183	0.000	0.218
**1**	0.042	10.966	16	10.943	0	87.543	1.002	1.091	0.088	0.104	0.199	0.096	0.569
**1**	0.033	9.213	24	0	0	156.301	0.693	0.679	0.014	0.108	0.113	0.006	0.285
**2**	0.033	6.100	18	0	0	177.049	0.485	0.477	0.009	0.209	0.217	0.008	0.133
**2**	0.050	16.530	49	0	3.630	177.858	0.488	0.589	0.102	0.105	0.092	0.013	0.249
**2**	0.033	10.370	26	0	0	150.434	0.609	0.633	0.024	0.227	0.200	0.027	0.201
**2**	0.040	6.892	25	0	0	217.644	0.431	0.443	0.012	0.133	0.121	0.012	0.155
**2**	0.067	7.524	38	0	0	303.030	0.326	0.326	0.000	0.069	0.077	0.007	0.128
**2**	0.042	15.777	22	0	3.803	83.666	0.774	0.758	0.016	0.155	0.164	0.009	0.295
**2**	0.033	9.844	24	0	0	146.282	0.591	0.606	0.015	0.245	0.231	0.014	0.180
**2**	0.033	8.267	22	0	0	159.671	0.603	0.585	0.018	0.197	0.200	0.003	0.195
**2**	0.033	7.974	20	0	0	150.489	0.643	0.622	0.021	0.169	0.201	0.032	0.225
**2**	0.033	6.400	18	0	0	168.750	0.596	0.554	0.042	0.148	0.189	0.041	0.198

**Table 3:** Infant walkers gait characteristics description of functional and temporal parameters.

Group	Novice N = 10			Improver N = 10			Mann-Whitney U	Z	Sig. *σ* (2-tailed)	Effect size *r*
Parameters	Median (Interquartile Range)	Mean Rank	Sum of Ranks	Median (Interquartile Range)	Mean Rank	Sum of Ranks				
**FALLS [falls/min]**	1.79 (9.90)	13.00	130	0.00 (0.00)	8.00	80	25	-2.48	0.01*	0.56*
**STOPS [stops/min]**	1.34 (3.90)	12.40	124	0.00 (0.90)	8.60	86	31	-1.62	0.11	0.36
**CAD [steps/min]**	98.12 (87.68)	7.10	71	164.21 (38.41)	13.90	139	16	-2.57	0.01*	0.57*
**ST_R [s]**	0.80 (0.43)	14.20	142	0.59 (0.15)	6.80	68	13	-2.80	< 0.01*	0.63*
**ST_L [s]**	0.79 (0.53)	13.80	138	0.59 (0.16)	7.20	72	17	-2.50	0.01*	0.56*
**ST_Diff _ΔR-L_ [s]**	0.04 (0.07)	11.60	116	0.02 (0.02)	9.40	94	39	-0.83	0.41	0.19
**SW_R [s]**	0.14 (0.07)	9.10	91	0.16 (0.09)	11.90	119	36	-1.06	0.29	0.24
**SW_L [s]**	0.15 (0.08)	8.50	85	0.19 (0.09)	12.50	125	30	-1.51	0.13	0.34
**SW_Diff _ΔR-L_ [s]**	0.02 (0.04)	11.50	115	0.01 (0.02)	9.50	95	40	-0.76	0.45	0.17
**DS [s]**	0.34 (0.37)	14.60	146	0.20 (0.08)	6.40	64	9	-3.10	< 0.01*	0.69*

FALLS, frequency of falls; STOPS, frequency of stops; CAD, gait cadence; ST, stance phase time; SW, swing phase time; DS, double support phase time; R, right; L, left; Diff, difference between legs

* *p* ≤ 0.05.

## Discussion

This first non-laboratory investigation of infant´s gait in their home environments has described how infants move in familiar environments and identified significant differences in functional and temporal gait parameters between novice and improver walkers. Consistent with the hypothesis, specific differences included a reduced frequency of falls in more experienced walkers (functional), and in terms of temporal aspects an increase in gait cadence, and a decrease in stance phase time and double support time, all with large effect sizes.

Analysis of the video data in the current study showed that functional characteristics of infant gait in real-world environments differ dependent on their walking experience, specifically there was a decreasing frequency of falls with increasing gait experience during infant walking. Alongside this change in falls we would have anticipated a reduction in stops as the infants become more experienced walkers, however this was not the case. This may be influenced by the definition of a stop in the study being longer than 4s, which could make it difficult to find statistical differences between the two groups. However, slow transitions to the alternate leg in our sample lasted approximately 2 s, thus, we assumed pauses longer than 4 s were an appropriate definition. Furthermore, the nature of the data sourcing, inclusion criteria and analysis in the current study allowed infants to stop, turn or fall just as a result of natural movement consequences as we analysed open-source video and did not apply a protocol to the data collection. Distractions or other occurrences within the home environment might also be the reason for infants stopping or falling, on the other hand. The increasing confidence of the more experienced walkers in our research is also evidenced by their ability to carry objects and tendency to walk in self-motivated situations without being encouraged (Table 1). This contrasts the novice walkers, with less walking experience, who when self-selecting tasks opted not to be mobile and only one of whom was carrying an object compared to four in the improver group. The falls data in the current study is in accordance with literature collected in laboratory based playroom, which was validated to behaviour at home [13] that more experienced walkers will walk more and falls less frequently, although authors have compared slightly older infants (12–19 months of age) than in the current study (7–13 months of age). No difference in number of steps per hour or number of falls per hour were found between the laboratory and home measures (*t* tests comparing home (n = 15) and laboratory (n = 70) observations of infants with equivalent walking age) [13]. However, authors did not test the same infant at home and at the playroom styled laboratory. Furthermore, as it was more psychologically focused study, the study did not capture and compare other infant´s gait characteristics which could be influenced by an unfamiliar laboratory environment, for example, spatiotemporal gait parameters. The data sourcing and analysis in the current study has enabled the concurrent analysis and description of both functional and temporal measures in a familiar environment.

In addition to the functional differences identified between novice and improver walkers, this open-source data also enabled the quantification of temporal characteristics. The normal gait cadence data in literature shows comparable results to the results of the current study [8,22]. However, recent studies conclusions regarding infant´s gait cadence maturation are very often in contrast with each other. Some authors denote that cadence is constantly increasing with age and walking experience [10,23], while others show converse results [22]. Owen [22] presents slightly higher values and only decreasing tendency of cadence during aging (180 steps/min in 12 months old infants; and 171 steps/min in 18 months old infants) in comparison with the current study results of gait cadence (98 steps/min in novice walker group, 7–12 months old, mean 8.9±1.58 months; and 164 steps/min in improver walker group, 8–13 months old, mean 11±1.48 months). This contrast could be attributed to the different age of investigated infants and by the difference between laboratory and familiar environment measurements. Futhermore, long stops during walking bouts reduced with age and walking experience as well, which may have inflated the difference in the cadence values between the two groups in the current study. Our study is in confirmation with longitudinal observation study results of Bisi and Stagni [8] who found firstly increasing gait cadence in novice walkers and then decreasing cadence of improver walkers (140 steps/min in the onset of independent walking, then 178 steps/min after 2 months, and 148 steps/min after 6 months after walking onset). Although we obtained slightly lower values, our data reflects real and natural infant gait. All studies, including the current research, agree that during the early stage of walking (3–6 months after the onset of gait) the infant is just learning to maintain gait postural requirements in gravity, and, in the second stage, the infant is fine tuning their gait pattern [5,8,10,22,23]. However, from our results, we assume that in the very early phase of independent walking, the lower gait cadence values may dominate and, with more walking experiences, gradually increase towards higher values. When infants improve their gait in gravity and second phase starts, the gait cadence gradually decreases until the gait is finally tuned in adulthood. Although the days after walking on-set in our sample are undefined, the open-source video data analysed within this study potentially enabled the analysis of earlier steps than may have previously been quantified in infant walking in laboratory environments. However a longitudinal reasearch study recruiting and including infants from gait onset would enable these contradictions to be clarified.

Further temporal characteristics of infant novice and improver gait differed as significant difference in stance phase time between the two have been presented in our results. Novice infant walkers have longer stance phase duration (0.80 s for right leg, and 0.79 s for left leg) in comparison with more mature improver walkers (0.59 s for both legs). In comparison to other literature collected in laboratory environments the mature improver walker values are most consistent with those reported as adopting gait 0.52 s SD 0.11 [17] and within the first week of independent walking 0.45 IQR 0.22 [24]. This stance phase related finding contrasts the natural maturation in walking pattern of single leg stance described in existing literature: the more experienced walker the longer single leg stance phase duration [25,26] until a level equivalent to that of a normal adult is reached, by 3.5–4 years of age [26]. As aforementioned, the methodology in which the current study defined stopping may have elevated stance phase time values, particularly for the novice walkers therefore explaining this contradiction with existing literature and the large IQR in this variable in the current study.

The novice infants in this study had a longer single leg stance time coupled with a longer double support time to maximise stability in this early stage of gait [25]. Double support time of infant decreasing with age and walking experience is reported in previous research undertaken in laboratory environments [10,25,27]. Through maximising stability in this manner novice walkers lose propulsion [27], hence the lower cadence identified in this study. Interestingly, Clark and Phillips [27] refer that early walkers increase their time in double support during stance phase from 20% (adult referral value) up to nearly 60%. In our research sample, novices spent 42.5% of stance phase in the double support and improvers 33.9%. These values are slightly lower than the referred above, however, the decreasing trend towards adult values is depictable within the familiar environment data set in this study.

Current results show greater inter-individual variability in novice in comparison with improver walkers in stance phase time and double support time (Fig 2). However, in gait cadence, improvers presented more variability which could be explained by the early and not yet finished maturation of gait cadence in some infants. Additionally, the variable environments and aforementioned interactions with parents, pets etc may have impacted on the number of steps the individuals took, particularly in the improver group. The methodology of collection and analysis of open-source video means that the descriptive characteristics relating to the participants, such as days since first steps, are not clearly defined or reported and the videos defined by our raters as “improver” walkers may in fact be infants who are yet to increase their cadence as much as others within their cohort.

### Limitations

This study has some limitations largely due to the open-source nature of the videos. Firstly, the study has been undertaken with a relatively small sample number due to inclusion criteria which limited number of available videos significantly. This low availability of videos has also led to a lack of standardisation of video; with the environment such as other individuals, flooring and room size varying between videos. Secondly, due to the nature of the data in this research study it can only provide limited functional and temporal kinematic gait data. The size effect i.e. lower limb size in the obtained data was not possible control as limb size could not be effectively computed from the video with any certainty due to different perspectives and zoom/resolution of data participants. The data accuracy may also be influenced by the parent´s self-reporting of age and additional information, the quality of non-professional video recording, video resolutions, and inconsistent range of video sampling rates. These ranged between 0.033 Hz and 0.67 Hz with the following distribution of various frame rates: 0.033 Hz (n = 13); 0.040 Hz (n = 2); 0.042 Hz (n = 2); 0.050 Hz (n = 1); and 0.067 Hz (n = 2) with almost equal distribution in both groups. Additionally, there is a lack of information about the infant participant such as a birth date, health condition details, or basic anthropometry which limits the generalisability of the sample and would be available in a standard gait laboratory study. Similarly, the videos of the infants may have been recorded and published due to them walking early for example or undertaking a specific task which might lead to task specific outcomes, and therefore their relevance to the wider population of walking infants is unclear and undefined.

## Conclusions

Our study is the first to investigate infant´s functional and temporal gait parameters in their home environments using open-source gait data. Analysed data presented significant differences between novice and improver walkers in falls frequency, gait cadence, stance phase time, and double support phase time. The recent study results can serve as basic, but limited, data set for further research, for example, external validation of some laboratory protocols for infants. There are clear challenges with collection and interpretation of, and long-term access to, open-source gait data. However, it offers a rich availability for gait analysis in familiar environments of participants whose behaviour would otherwise be significantly influenced in a laboratory. Future research should be focused on familiar environment gait data collection in infants to identify their natural movement patterns more precisely consecutively with the psycho-social context.
